# Report of the Illinois State Board of Health

**Published:** 1884-09-20

**Authors:** 


**Report of the Illinois State Board of Health.**—One of the most important publications issued of late years in our country comes from the State Board of Health of Illinois, which is constituted by law the Board of Medical Examiners for the State. The report is entitled, "Medical Education and the Regulation of the Practice of Medicine in the United States and Canada." It contains all the medical laws existing in the region designated; an account of every medical school, regular or irregular, setting forth the number of professors, matriculants, and graduates in each, with the requirements and fees; a list of Schools not recognized by the board; an analysis of the schools and graduates, showing the number from each State in every school, and other valuable information, the collection of which must have involved an immense amount of labor on the part of the Illinois Board. The number of physicians in the United States and Canada is 90,410, being one to 600 of the population. The proportion of physicians to population in Canada as here given is 1:1112; New Mexico, 1:1484; S. Carolina, 1:1085; Utah, 1:1035; N. Carolina, 1:1029. In all the other districts the proportion is 1 to less than 1000. According to the law of Illinois, the Board is required to license all physicians of whatever school, provided the school comes up to a certain standard in its course of study and other conditions. Following out this rule there are twenty-four institutions, eclectic, physio-eclectic, physio-medical, hygeo-therapeutic, and a strong sprinkling of Buchanan and other bogus institutions not recognized, the holders of such diplomas being refused license. We notice that no small proportion of these contraband schools are recognized by the Eclectic Board of California. One-seventh of all the eclectics licensed in the State of California were licensed on diplomas from the Oakland Eclectic School, and 47 of the 266 were licensed on examination by the Eclectic Board, one-third of all the licensed eclectics in California deriving their authority to practice from these two sources.—*Ex.*

---

ONE of the Astors has given the Cancer Hospital of N. Y. $200,000.

